# Modulation of UVB-induced Carcinogenesis by Activation of Alternative DNA Repair Pathways

**DOI:** 10.1038/s41598-017-17940-8

**Published:** 2018-01-15

**Authors:** Yan Sha, Vladimir Vartanian, Nichole Owen, Stephanie J. Mengden Koon, Marcus J. Calkins, Courtney S. Thompson, Zahra Mirafzali, Sara Mir, Lisa E. Goldsmith, Huaping He, Chun Luo, Scott M. Brown, Paul W. Doetsch, Andy Kaempf, Jeong Y. Lim, Amanda K. McCullough, R. Stephen Lloyd

**Affiliations:** 1 Oregon Institute of Occupational Health Sciences, Oregon Health & Science University, 3181 S. W. Sam Jackson Park Rd, Portland, Oregon 97239 USA; 2 Department of Dermatology, Oregon Health & Science University, 3181 S.W. Sam Jackson Park Rd, Portland, Oregon 97239 USA; 3 Encapsula NanoSciences, 6 Cadillac Dr, Suite 245, Brentwood, TN 37027 USA; 4 Accelagen Incorporated, 6044 Cornerstone Ct W Suite C, San Diego, CA 92121 USA; 5 Brown Hale Consulting, Inc. 10842 Loire Ave., San Diego, CA 92131 USA; 6 Department of Biochemistry and Radiation Oncology, Winship Cancer Institute, Emory University School of Medicine, Atlanta, GA 30322 USA; 7 Genome Integrity and Structural Biology Laboratory, National Institute of Environmental Health Sciences, National Institutes of Health, Durham, NC 27709 USA; 8 Knight Cancer Institute Biostatistics Shared Resource, Oregon Health & Science University, 3181 S.W. Sam Jackson Park Rd, Portland, Oregon 97239 USA; 9 Department of Molecular and Medical Genetics, Oregon Health & Science University, 3181 S.W. Sam Jackson Park Rd, Portland, Oregon 97239 USA; 10 Present Address: Institute for Occupational Health Science, Shenzhen Prevention and Treatment Center for Occupational Diseases, 2019 Buxin Rd., Luohu District, Guangdong Province Shenzhen, 518020 China; 11 Present Address: Institute of Cellular and Organismic Biology, Academia Sinica, 128 Academia Road, Sec. 2, Nankang, Taipei 11529 Taiwan

## Abstract

The molecular basis for ultraviolet (UV) light-induced nonmelanoma and melanoma skin cancers centers on cumulative genomic instability caused by inefficient DNA repair of dipyrimidine photoproducts. Inefficient DNA repair and subsequent translesion replication past these DNA lesions generate distinct molecular signatures of tandem CC to TT and C to T transitions at dipyrimidine sites. Since previous efforts to develop experimental strategies to enhance the repair capacity of basal keratinocytes have been limited, we have engineered the N-terminally truncated form (Δ228) UV endonuclease (UVDE) from *Schizosaccharomyces pombe* to include a TAT cell-penetrating peptide sequence with or without a nuclear localization signal (NLS): UVDE-TAT and UVDE-NLS-TAT. Further, a NLS was engineered onto a pyrimidine dimer glycosylase from *Paramecium bursaria* chlorella virus-1 (cv-pdg-NLS). Purified enzymes were encapsulated into liposomes and topically delivered to the dorsal surface of SKH1 hairless mice in a UVB-induced carcinogenesis study. Total tumor burden was significantly reduced in mice receiving either UVDE-TAT or UVDE-NLS-TAT versus control empty liposomes and time to death was significantly reduced with the UVDE-NLS-TAT. These data suggest that efficient delivery of exogenous enzymes for the initiation of repair of UVB-induced DNA damage may protect from UVB induction of squamous and basal cell carcinomas.

## Introduction

Non-melanoma skin cancers (NMSCs), including basal cell carcinoma (BCC) and squamous cell carcinoma (SCC) are the most prevalent types of human cancers, affecting over five million people in the United States annually, and costing billions of dollars for health care and loss of work^1–3^. In addition to high rates of disease in the general population, organ transplant patients have a greater than 50-fold increase in the incidence of NMSC, with increased risk of metastasis^4–6^. Current methods for treatment of NMSC, including surgical resection of the tumor, are associated with considerable pain and morbidity. Given these exceptionally high incidence rates, strategies to prevent skin cancer have predominantly focused on recommendations for sun avoidance, restricted access of youth to tanning beds, the use of broad spectrum UVA and UVB sunscreens, and application of topical anti-oxidants. However, these recommendations have not sufficiently diminished the prevalence of NMSC, and development of novel methods to reduce or prevent NMSCs would not only alleviate suffering, but also substantially reduce health care costs.

Exposure to UV irradiation in sunlight causes NMSC by inducing DNA damage that if replicated, leads to mutations in genes such as *KRAS* or *TP53*^7^. Two common types of UVB-induced DNA damage are cyclobutane pyrimidine dimers (CPDs) and 6–4 photoproducts (6–4 PPs). 6–4 PPs are more rapidly repaired than CPDs in both fibroblasts and keratinocytes^8^. In response to broad-spectrum UV-induced DNA damage, skin cells transiently arrest progression through the cell cycle to allow DNA repair or, in the case of irreparable DNA damage, die via apoptosis. CPDs account for at least 80% of UVB-induced mutagenesis but contribute only modestly to cytotoxicity in a mammalian cell model; however, 6–4 PPs are minimally mutagenic but highly cytotoxic^9,10^. Thus, it can be concluded that coordination of these responses is crucial for protection against skin carcinogenesis.

Humans possess only one mechanism, the nucleotide excision repair (NER) pathway, for repairing dipyrimidine DNA lesions. The importance of this DNA repair system in limiting NMSC is best demonstrated by the clinical sequela of patients suffering from the autosomal recessive genetic disorder Xeroderma Pigmentosum (XP). These patients have defects in either NER or DNA translesion synthesis (TLS) of CPDs, either of which will significantly increase the risk for development of NMSC. In fact, XP patients have greater than a 1000-fold increased risk of developing skin cancer before the age of 20 compared to the general population^11^.

In contrast to humans, some organisms can utilize the base excision repair (BER) pathway in addition to NER, for repairing UV-induced DNA lesions. Humans have all of the enzymes necessary for completing BER, but lack an enzyme, a pyrimidine dimer-specific DNA glycosylase (pdg) to recognize CPDs and initiate the cascade. Therefore, one strategy for enhancing repair of CPDs in human skin cells has been to deliver, or express, the bacteriophage T4 pdg in these cells^12^. Collectively, several studies have shown that T4-pdg could not only initiate repair following UV damage of XP cells^13^, but also increase survival in XP cells^14^. Further, T4-pdg protein has been encapsulated into a liposomal delivery vehicle for use in studies on murine and human skin^15,16^. Delivery of T4-pdg in mouse models increased the rate of CPD removal, reduced the frequency of SCC, and minimized UVB-induced immune suppression. Results of clinical trials with XP patients using topically delivered T4-pdg showed that new pre-cancerous lesions (actinic keratosis) were reduced by 68% and new cases of BCC were reduced by 30% compared to patients treated with placebo lotion^17^.

Although these data were encouraging, a potential limitation for the use of any pdg is that their substrate specificities do not include recognition of 6–4 PPs. However, the UV endonuclease (UVDE) from *Schizosaccharomyces pombe* has a very broad substrate specificity which includes both CPDs and 6–4 PPs and is known to initiate the nucleotide incision repair (NIR) pathway^18,19^. To increase its solubility while retaining full catalytic activity, previous studies have characterized a soluble form of UVDE in which the N-terminal 228 amino acids were deleted (Δ228-UVDE). Since the truncated form of UVDE was exclusively used in all studies described herein, it is abbreviated UVDE, but refers to Δ228-UVDE. Although this enzyme has not been previously used for *in vivo* repair and carcinogenesis studies, the increased substrate specificity makes it an ideal candidate for use in investigations of UV-induced carcinogenesis. Further limitations of the original study design with T4-pdg were that it poorly localized to the nucleus and once delivered to the skin, could not redistribute to cells in the immediate vicinity. To address these challenges, part of our experimental strategy was to add a cell-penetrating peptide from HIV Tat transcriptional activator (TAT)^20^ that facilitates migration of the associated protein between cells and to engineer a nuclear localization signal (NLS) onto the repair enzyme. Additionally, we engineered the cv-pdg enzyme to be expressed with a C-terminal NLS. Thus, the goals of this investigation were to test whether topical delivery of TAT- or NLS-TAT-modified UVDE or cv-pdg-NLS would differentially modulate UVB-induced carcinogenesis in a SKH1 hairless mouse model.

## Results

### Preparation of Dipyrimidine DNA Repair Enzymes for Topical Delivery

**Figure 1:**
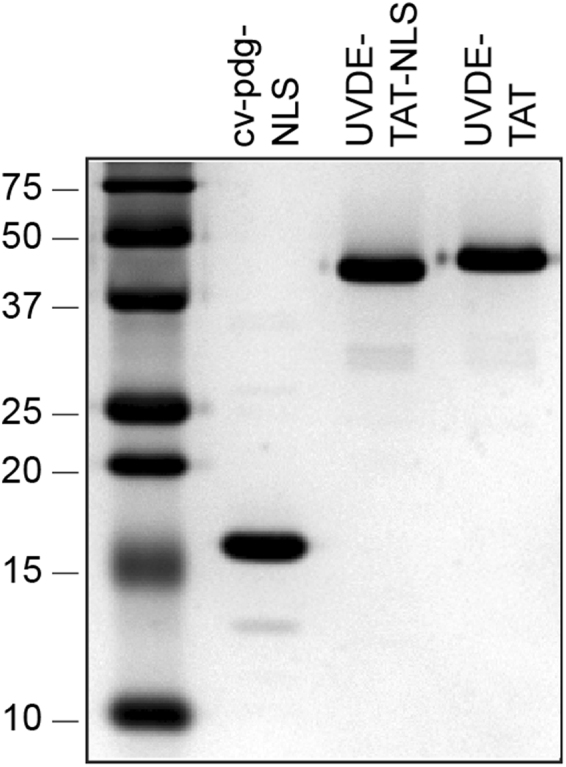
Purity of UVDE-TAT, UVDE-NLS-TAT, and cv-pdg-NLS. The purity of the DNA repair enzymes was assessed by Coomassie staining of proteins following electrophoretic separation through a denaturing 15% polyacrylamide gel. A total of 5 µg of UVDE-TAT, UVDE-NLS-TAT, and cv-pdg-NLS were run in lanes as indicated. Pre-stained markers were run in the left lane, with molecular weights given.

Since mammalian cells exclusively use NER to initiate the repair of dipyrimidine photoproducts, the focus of this investigation was to determine if the induction of UVB-induced NMSCs could be significantly reduced relative to controls through the delivery of an enzyme that repairs both CPDs and 6–4 PPs. To facilitate and maximize cellular delivery of UVDE, a sequence encoding a protein transduction peptide, TAT (YGRKKRRQRRR), was engineered onto the C-terminus of UVDE (UVDE-TAT). Further, since the catalytically-active, truncated form of UVDE lacks its natural NLS site, the *E. coli* expression vector was also modified to insert the 7-amino acid NLS (PKKRKRR) at the C-terminus (UVDE-NLS-TAT). The sequences of the complete genes were confirmed prior to expression studies. Protein expression and purification were optimized, with a final yield of UVDE-TAT and UVDE-NLS-TAT of 106 mg/L and 14 mg/L, respectively. Further, the expression construct for cv-pdg was engineered to contain the same 10 amino acid NLS on the C-terminal portion of the enzyme and purified, with the final yield being 55 mg/mL. The purities of these enzymes are shown in Fig. 1.

To topically deliver these enzymes to mouse skin, each protein was encapsulated at 10 μg/mL into liposomes using a formulation previously described for the T4-pdg studies^21^. Details for liposomal preparation are given in the Methods section. All liposomes were formulated with hydrogel to yield a final concentration of 0.75%, which allowed uniform distribution and absorbance into the mouse skin.

### UVB-induced Carcinogenesis

A total of 40 SKH1 hairless mice were assigned to 4 treatment groups in the following experimental design: 1) empty liposome control, 2) UVDE-TAT, 3) UVDE-NLS-TAT, and 4) cv-pdg-NLS. An additional 10 mice were not treated with liposomes or UVB to assess frequencies of spontaneous skin lesions; however, these mice never developed any spontaneous skin tumors and are not included in further analyses. Beginning at 8 weeks of age, 0.2 mL of each liposomal formulation was uniformly applied to the dorsal skin of each mouse using a pre-moistened cotton swab. This amount was absorbed to apparent dryness within 3–5 min after application. After 1 hr, mice were exposed to increasing doses of UVB irradiation in an 8-compartment chamber that was covered with a quartz plate to allow full UVB penetrance. This chamber maximized exposures to the backs of the mice.

**Figure 2:**
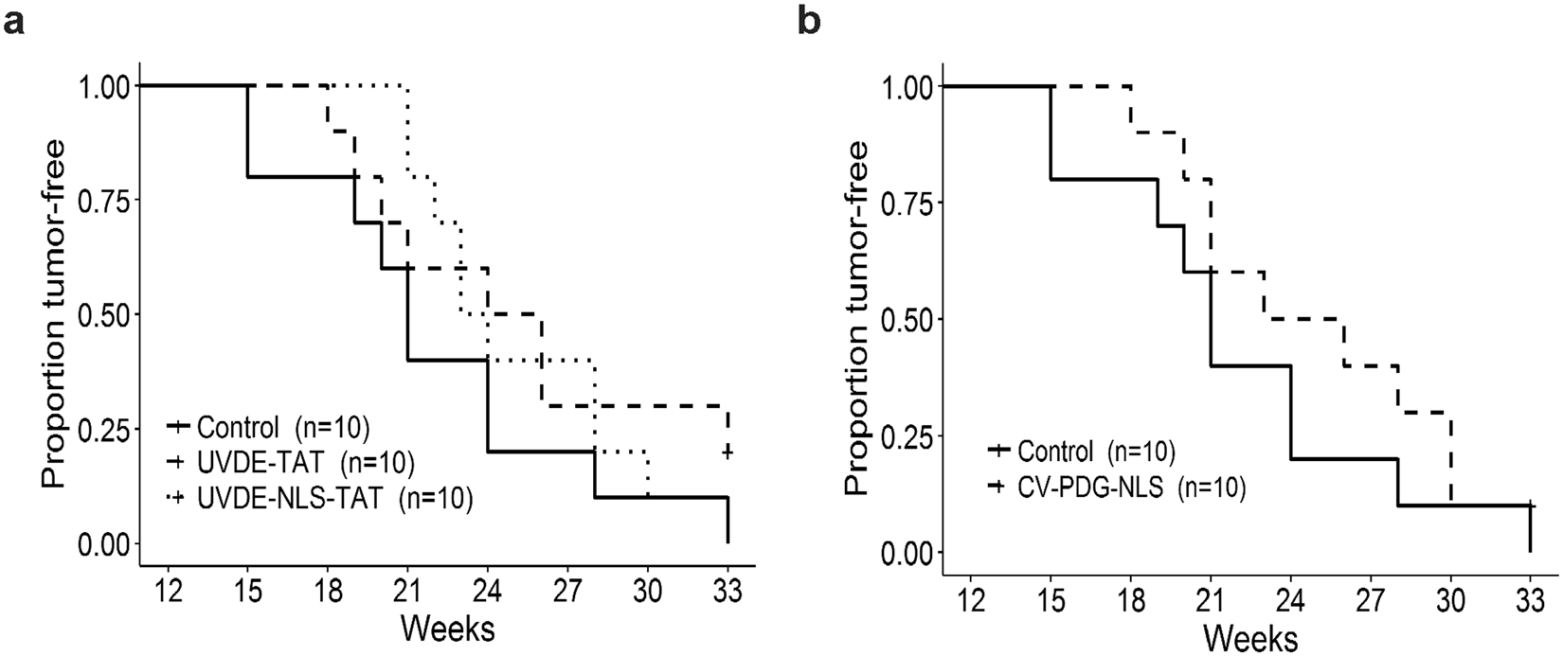
Proportion of tumor-free mice. SKH1 hairless mice were treated with control empty liposomes or liposomes containing either UVDE-TAT, UVDE-NLS-TAT, or cv-pdg-NLS 1 hr prior to irradiation. Mice were irradiated in individual chambers covered with quartz glass that allowed full UVB transmission of the increasing UVB doses delivered on a M, W, F schedule. Panels (**a**) and (**b**) show the proportion of mice in each group that have not developed a 2 mm tumor at specified time points across the study period. Panel (**a**) Control empty liposome formulation (solid line), liposomes containing UVDE-TAT (dashed line), and liposomes containing UVDE-NLS-TAT (dotted line). Panel (**b**) Control empty liposome formulation (solid line) and liposomes containing cv-pdg-NLS (dashed line). Proportions of tumor-free mice in each group were plotted with Kaplan-Meier curves and compared using the log-rank test. Mice that never developed a 2 mm tumor during the study period were represented as plus (+) signs on the plots. Severe ulceration meeting the criteria for mandatory euthanasia never occurred for tumors ≤2 mm.

To prevent significant skin irritation and burning, a UVB dose escalation strategy was used for the initial irradiations. As anticipated, following 9 UVB exposures (a total exposure of 2.0 kJ/m^2^) over the course of 3 weeks, the dorsal skin showed evidence of mild redness, slight loss in elasticity, and thickening, without any evidence of sunburn. All mice continued to receive average weekly doses of ~22 kJ/m^2^ and all mice were monitored three times per week for skin lesions. The earliest time for the formation of confirmed squamous cell carcinomas in the control empty liposome group was at 15 weeks, with a total cumulative exposure of ~217 kJ/m^2^_._ Analyses of the length of time to form the first tumor (≥2 mm diameter) in each irradiated mouse revealed that although there were trends for a delay in time to first tumor in all 3 active enzyme groups (shown in Fig. 2a,b and quantified by the median in Table 1), these delays were not statistically distinguishable from the control group, with nonsignificant p-values of 0.404 for UVDE-TAT, 0.788 for UVDE-NLS-TAT, and 0.668 for cv-pdg-NLS.

**Table 1:** Time to First 2 mm Tumor (weeks) Summary Statistics.

Treatment group	Group Size (# mice)	# mice with a 2 mm tumor	**Range** of times to first 2 mm tumor	**Median** time to first 2 mm tumor (95% CI)
Control	10	10	15 to 33 weeks	21 weeks (15.0 to 24.0)
UVDE-TAT	10	8	18 to 33 weeks	25 weeks (18.0 to 33.0)
UVDE-NLS-TAT	10	10	21 to 33 weeks	23.5 weeks (21.0 to 28.0)
cv-pdg-NLS	10	9	18 to 33 weeks	24.5 weeks (18.0 to 30.0)

Note: the sample median in each group is the same as the Kaplan-Meier estimated median because all mice were evaluated until euthanasia was required or the end of the study was reached (after 33 weeks of UVB).

**Figure 3:**
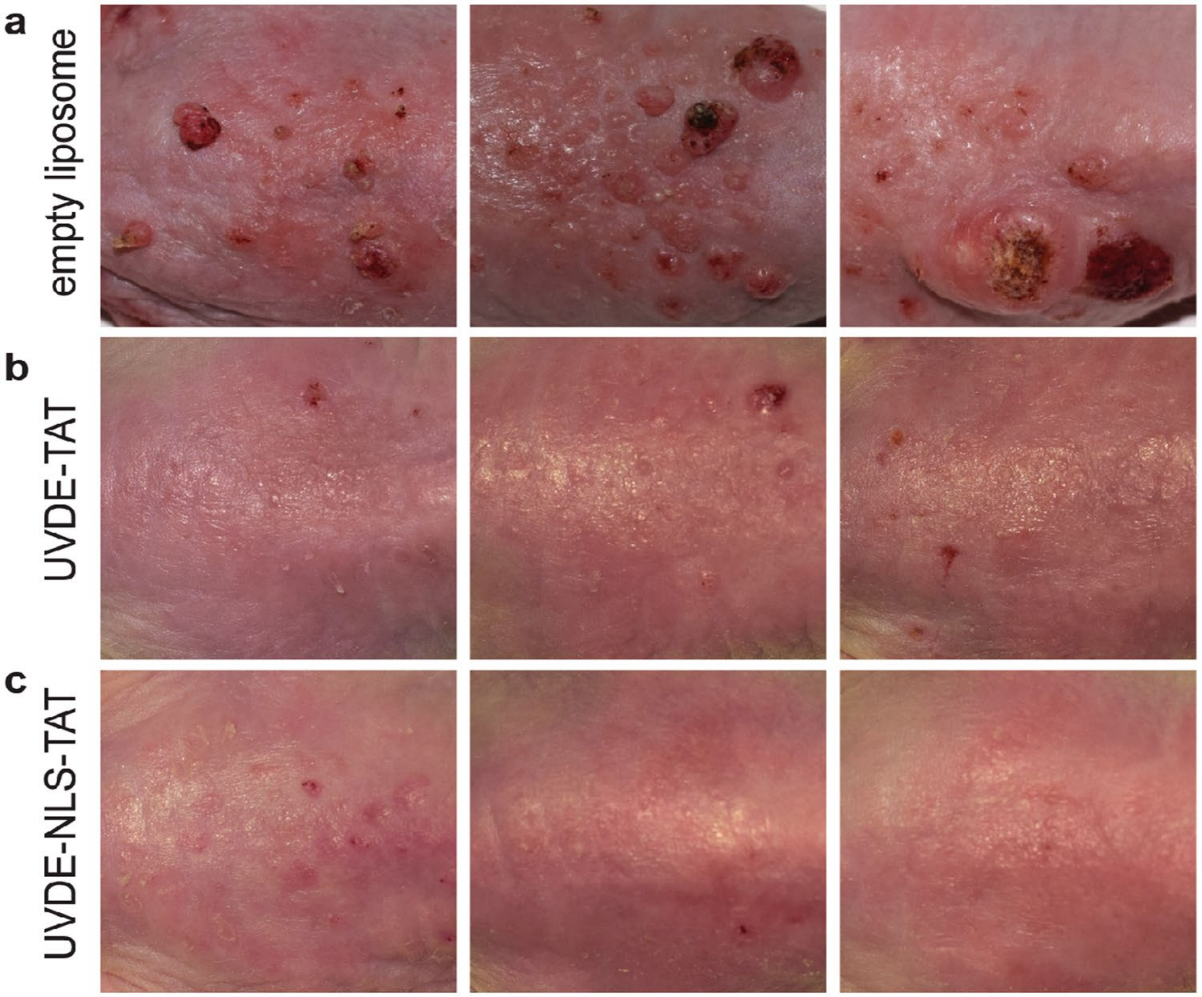
Suppression of tumor formation in SKH1 hairless mice treated with liposomes containing UVDE-TAT and UVDE-NLS-TAT. Three representative photographs of mice from each treatment group are shown: control empty liposome formulation Panel (**a**), liposomes containing UVDE-TAT Panel (**b**), and liposomes containing UVDE-NLS-TAT Panel (**c**) at 23 weeks (cumulative dose of 311 kJ/m^2^).

Following 23 weeks of UVB exposures, totaling 311 kJ/m^2^, all mice were photographed for comparative analyses. Representative images of mice treated with control empty liposome, UVDE-TAT, and UVDE-NLS-TAT are shown in Fig. 3, Panels a–c. Visual evaluation of the mice treated with control empty liposomes versus any of the enzyme-containing liposomes revealed a greater involvement of the dorsal surface area with various tumors, with 4 of the 10 mice having tumors that were >3 mm. In contrast, mice treated with liposomes containing an enzyme showed less severe damage, and no tumors >3 mm observed in either form of UVDE, and only one tumor >3 mm in the cv-pdg-NLS group. These observations indicated a suppressive effect on tumor formation with the treatment of either of the repair enzymes.

**Figure 4:**
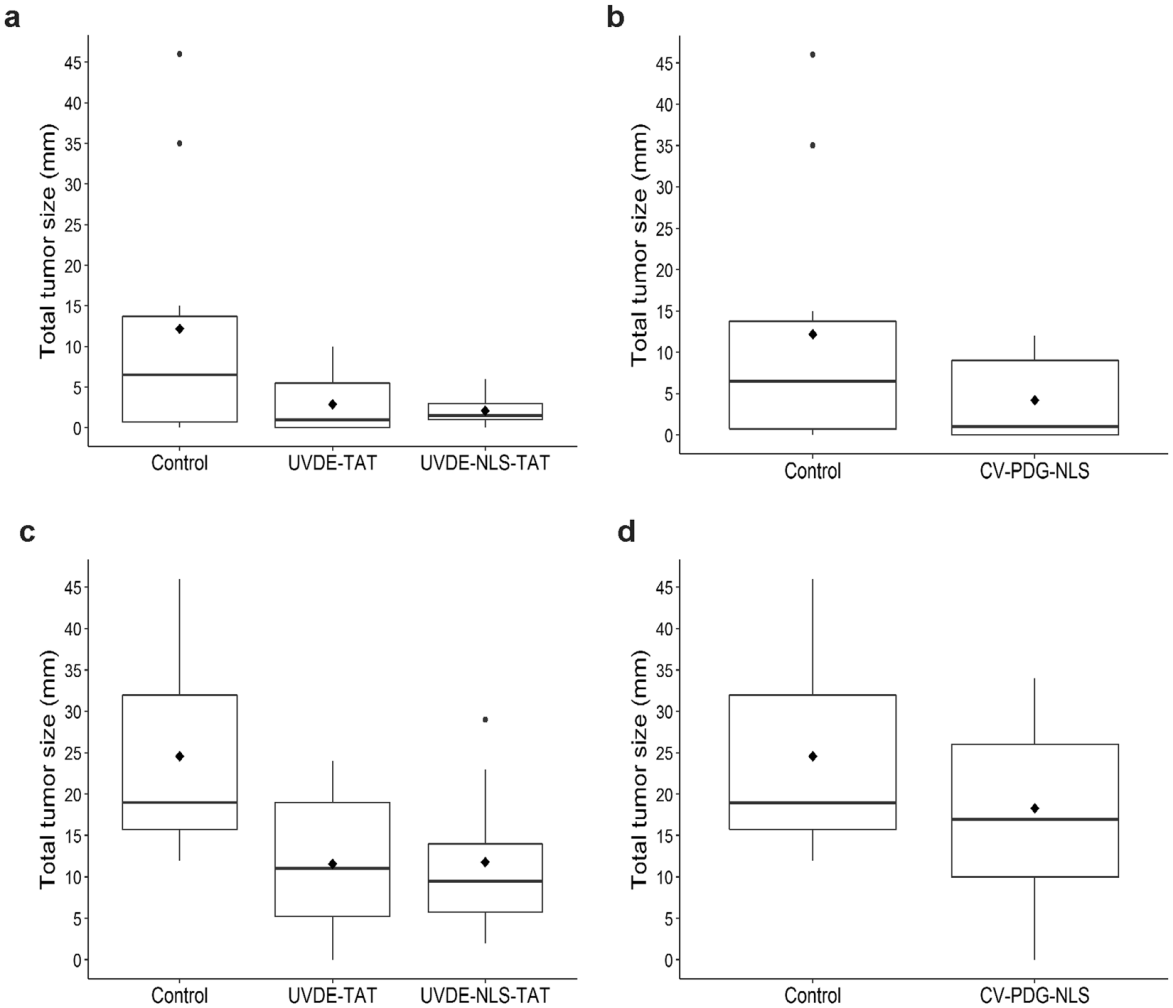
Analyses of total tumor size at 23 and 33 weeks of UVB irradiation. To assess the extent of protection afforded by topical delivery of UVDE-TAT and UVDE-NLS-TAT Panels (**a**) and (**c**) and cv-pdg-NLS Panels (**b**) and (**d**), respectively, analyses of the aggregate size of UVB-induced tumors for each mouse are shown for data collected at 23 Panels (**a**) and (**b**) and 33 Panels (**c**) and (**d**) weeks of irradiation. All tumors were measured and the sum of all tumor diameters was calculated for each mouse to represent total tumor burden. Final measured (pre-death) tumor sizes were used for mice that required euthanasia prior to weeks 23 or 33. At each time point of interest, the sample distributions of total tumor size for each of the 4 groups of mice is displayed as a box plot with a horizontal line inside the box depicting the median, a diamond symbol denoting the mean, and outliers represented as circles outside the box. Group comparisons of total tumor burden at each of the two time points were conducted using analysis of variance (ANOVA).

The total tumor burden (cumulative size of all tumors) for each mouse was analyzed at various time points, with the data for week 23 summarized in group-specific box plots for UVDE-TAT and UVDE-NLS-TAT (Fig. 4a) and for cv-pdg-NLS (Fig. 4b). At 23 weeks, the mean total tumor size per mouse for the empty, UVDE-TAT, UVDE-NLS-TAT, and cv-pdg-NLS liposome-treated groups were 12.2 mm, 2.9 mm, 2.1 mm, and 4.2 mm, respectively. The mean differences in total tumor size for UVDE-TAT, UVDE-NLS-TAT, and cv-pdg-NLS relative to the control group were not statistically significant (*p* = 0.092, 0.058, and 0.179, respectively). The empty-liposome treated group had a total of 28 tumors with an aggregate total tumor size of 122 mm at 23 weeks. In contrast, the UVDE-TAT, UVDE-NLS-TAT, and cv-pdg-NLS groups had 18, 15, and 19 tumors, respectively, and aggregated tumor sizes of 29 mm, 21 mm, and 42 mm, respectively. The 23-week time point was chosen for analyses because only 1 mouse (in the control empty liposome treated group) had developed a tumor that required the mouse to be euthanized. In this, and subsequent analyses of total tumor burden, mice that were euthanized prior to a specified time point were assigned tumor size values equal to their last measurements while alive. However, we acknowledge that using the final observed values of euthanized mice for these analyses is an underestimate of total tumor burden as it does not account for the anticipated tumor growth that would likely have continued to occur in these animals. Imputation of these final measurements at post-death time points was important since exclusion of euthanized mice would bias the total tumor size comparisons, since mice with the largest tumor burdens (>8 mm for a single tumor) would be unavailable for the between-group analyses.

Comparable analyses were also made at 33 weeks with these data summarized in Fig. 4c,d. The mean total tumor size per mouse was ~25 mm in the control group, ~12 mm in both the UVDE treatment groups, and 18.3 mm in the cv-pdg-NLS group. These tumor burden averages, when compared to empty liposome controls, were statistically significant for UVDE-TAT (*p* = 0.041) and UVDE-NLS-TAT (*p* = 0.045), but not for cv-pdg-NLS (*p* = 0.541). These findings demonstrate that, relative to the control empty liposomal treatment, application of liposomes containing UVDE-TAT or UVDE-NLS-TAT reduces the UVB-induced total tumor burden.

**Figure 5:**
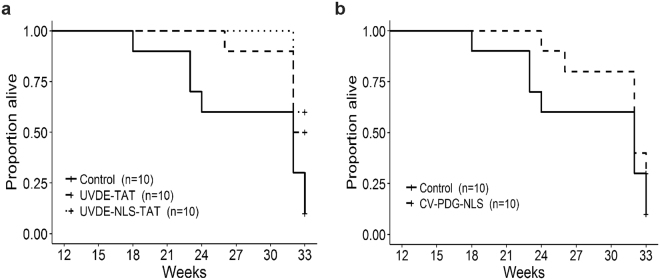
Kaplan-Meier plot of survival. Throughout the course of UVB irradiation, mice were routinely monitored for tumors that became ulcerated or were >8 mm in diameter. Mice that met either of these criteria were euthanized, which served as the death endpoint for survival analysis. The proportion of mice not developing a tumor that met the above criteria is plotted across the study period. Panel (**a**) control empty liposome formulation (solid line), liposomes containing UVDE-TAT (dashed line), and liposomes containing UVDE-NLS-TAT (dotted line). Panel (**b**) control empty liposome formulation (solid line) and liposomes containing cv-pdg-NLS (dashed line). The plotted survival proportions were estimated using the Kaplan-Meier method and statistically compared with the log-rank test. Mice that did not meet the criteria for euthanasia by the end of the 33-week study period (i.e., censored observations) were represented with plus (+) signs. Since all mice were analyzed until either (i) euthanasia due to tumor criteria or (ii) the study ended, there was no censoring prior to the last observed time point at 33 weeks and Kaplan-Meier estimates were equal to the sample proportions of mice remaining alive at any given time.

In addition, analyses were performed concerning time-to-death for each of the groups. To compare the risk of tumor-induced euthanasia for the three active enzyme groups relative to the control group, hazard ratios (HR) were estimated from a Cox regression model. Assuming proportional risks of euthanasia across all time points, estimated hazard (risk) ratios involving the control group were 0.35 (95% CI: 0.11, 1.05) for UVDE-TAT, 0.25 (95% CI: 0.07, 0.81) for UVDE-NLS-TAT, and 0.56 (95% CI: 0.21, 1.55) for cv-pdg-NLS. Thus, UVDE-NLS-TAT mice had 75% less (95% CI: 93% to 19% less) risk of being euthanized than control mice during the treatment period. Without having to assume a constant risk ratio across time, analyses of Kaplan-Meier survival curves of control vs UVDE-TAT and UVDE-NLS-TAT mice revealed survival advantages in these active treatment groups, with log-rank test *p* values of 0.135 and 0.037, respectively (Fig. 5a). A comparable analysis of another encapsulated enzyme compared to controls (Fig. 5b) revealed less of a survival advantages for cv-pdg-NLS (*p* = 0.598) than was observed for the two UVDE groups.

**Figure 6:**
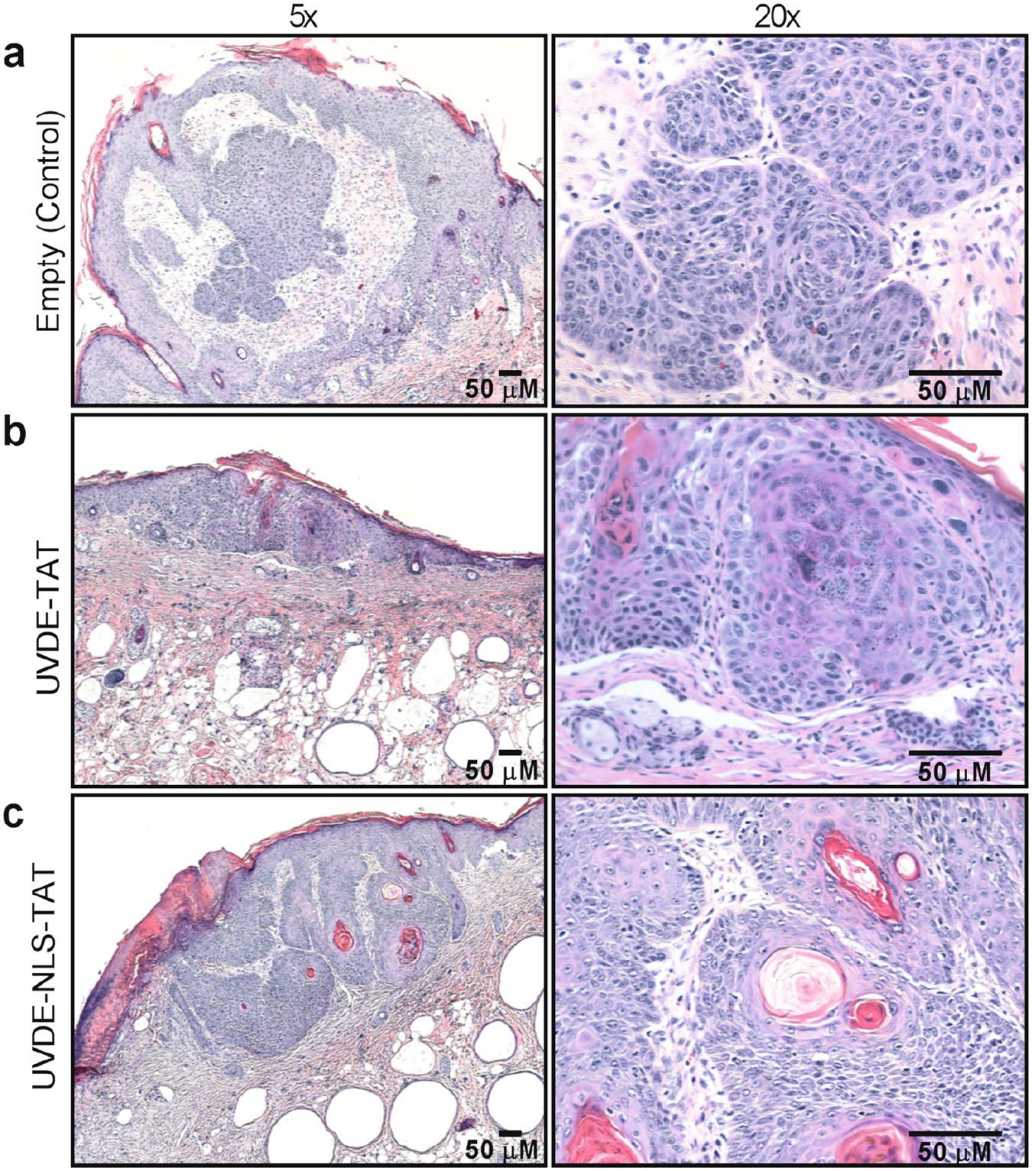
Representative histology of tumors formed following UVB irradiation. At the time of euthanasia, representative skin tissue samples were harvested, prepared for histologic analyses, and photographed at 5 and 20x in left and right panels, respectively. Representative tumors are shown from skin tissues containing tumors from the following groups: control empty liposomes Panel (**a**), liposomes containing UVDE-TAT Panel (**b**), and liposomes containing UVDE-NLS-TAT Panel (**c**). No qualitative differences were noted in the general characteristics of the tumors that were formed as a result of the cumulative UVB irradiations. All inset bars represent 50 µM.

At the time when mice were euthanized, skin strips were harvested and processed for H&E staining. Even though the total tumor burden at 33 weeks and the time-to-death analyses revealed significant differences, histologic analyses of the tumors that formed were indistinguishable among the 4 groups. All examined tumors were asymmetric and poorly circumscribed epithelial neoplasms, characterized by irregular aggregates extending into the superficial to deep dermis or, in some cases, into the subcutis and underlying muscle (Fig. 6). Atypical keratinocytes had enlarged, hyperchromatic and pleomorphic nuclei, were undergoing mitoses or apoptosis, and exhibited variable levels of eosinophilic cytoplasms. These features are highly characteristic of SCCs.

## Discussion

The temporal hierarchy of NER of UV-induced dipyrimidine photoproducts in mammalian cells is characterized by not only rapid recognition and excision of 6–4 PPs (which are preferentially formed in open chromatin regions), but also preferential repair of CPDs in actively transcribed genes^22–24^. The remaining dipyrimidine photoproducts are repaired at greatly reduced rates that can extend over several days following a UV dose equivalent to a minimal sunburn. If cytosine bases are at either the 5′ or 3′ position or both, these damages can undergo spontaneous deamination to uracil that is highly mutagenic if replicated. CPDs remaining in the genome also function as one of the main causes of UV-induced immune suppression^25,26^. Thus, the potential deleterious effects of unrepaired DNA damage have the capacity to increase genomic instability and reduce immune surveillance, both of which increase the risk of cellular transformation.

A strategy to mitigate these issues is to enhance DNA repair capacity in damaged cells by activating an alternative DNA repair pathway. Since less complex organisms possess alternative pathways to repair pyrimidine dimers, such as photoreactivation, BER, and NIR, the methodological challenge is to efficiently deliver sufficient levels of a repair enzyme to activate analogous alternative pathways in mammals. In the case of photoreactivation, there is not only the issue of delivering CPD- and 6–4 PP-specific photolyases, but also establishing conditions required to irradiate with sufficient amounts of appropriately tuned wavelengths of visible light. To avoid the problems associated with photoreactivation, the delivery of CPD-specific DNA glycosylase/AP lyases to initiate BER, or the introduction of UV endonucleases to activate NIR, offer feasible alternatives.

Differences in substrate specificities allow each enzyme to provide specific advantages, with pdgs recognizing only CPDs and ring-fragmented purines, and UVDEs removing both 6–4 PPs and CPDs, but not the ring-fragmented purines. We anticipated that initiation of repair of both dipyrimidine photoproducts may have important biological implications. Since NEIL1- and OGG1-initiated repair of the ring-fragmented purines is likely to be sufficient to minimize the effects of these specific UVB-induced DNA lesions^27^, it is hypothesized that the repair of both types of dipyrimidine photoproducts leads to suppression of UVB-induced carcinogenesis.

Although not explicitly tested in this investigation, there are several potential mechanisms through which the topical delivery of UVDE could reduce the severity of UVB-induced carcinogenesis. These include: 1) increased, high-fidelity cellular DNA repair for CPDs and 6–4 PPs such that both dipyrimidine adducts are removed at an accelerated rate, thus reducing UVB-induced mutagenesis and carcinogenesis; 2) increased cell death in severely damaged cells via rapid initiation of single-strand breaks at CPDs and 6–4 PPs, thus killing potentially carcinogenic cells instead of undergoing error-prone replication; and 3) minimization of UV-induced immune suppression. Based on prior literature, we favor the mechanism of UVDE-initiated rapid repair of CPDs and 6–4 PPs in which the repair patch is anticipated to be synthesized with high fidelity. This conclusion is based on the following literature precedents. UVDE initiates repair at both dipyrimidine photoproducts by incision immediately 5′ to the damage^18,28,29^, followed by long-patch BER in conjunction with Rad27/Fen1, XRCC1, PARP1, and DNA polymerase^30–33^. Further, in UV-irradiated XPA cells expressing the *Neurospora crassa* UVDE, single-strand breaks were introduced immediately following irradiation and were efficiently repaired, resulting in enhanced survival approaching that of wild-type cells^31,32^. It was also demonstrated via expression of UVDE in repair-proficient and deficient *S. pombe* that introduction of the enzyme resulted in the initiation of repair of CPDs in both the nucleus and mitochondria, but that nuclear-localized repair was responsible for conferring enhanced survival in NER-deficient cells^34^. These mechanisms are inferred to occur via high fidelity reactions, since UVDE recognition of CPDs involves a quadruple flipping mechanism in which both the damaged dipyrimidine and the two complementary purines are extrahelical^35,36^.

Collectively, these data present consistent evidence that following UVB irradiation, UVDE-initiated repair results in rapid, long-patch processes, improving survival in DNA repair-deficient cells. The final product of long-patch BER is anticipated to be error-free since the undamaged complementary strand is available for repair-patch synthesis. This rapid repair of dipyrimidine photoproducts is also expected to greatly minimize UV-induced immune suppression since one of the primary signals for initiating this suppression is the duration of unrepaired CPDs in genomic DNAs^25,37–39^. This conclusion is also consistent with data from the XP clinical trial using T4-pdg in which suppression of new tumors could at least partially be explained by enhanced immune surveillance.

Germane to our study using cv-pdg-NLS, previous investigations led by Dr. Daniel Yarosh and colleagues at Applied Genetics, Inc. used a similar long-term carcinogenesis protocol in mice. They demonstrated that administration of liposomal-encapsulated T4-pdg, immediately following UVB irradiation, increased the mean time to first 1 mm tumor from 19.5 weeks for heat-inactivated enzyme to 21.8 weeks for the active enzyme^40^. Further, by 30 weeks of treatment, the number of tumors per mouse was ~30% less in the T4-pdg treated mice versus the control. This suppression in carcinogenesis was also enzyme concentration dependent. There are many distinctions between the Yarosh investigations and our current study including 1) differences between T4-pdg and UVDE in terms of catalytic efficiencies, stabilities, and substrate specificities; 2) T4-pdg being a native enzyme while the cv-pdg enzyme was engineered to contain a NLS; 3) concentration differences of the encapsulated enzymes, with Yarosh *et al*. administering T4-pdg at 1.0 μg/mL, while the current study used enzyme concentrations of 10 μg/mL; 4) the timing of UVB irradiation, which occurred prior to T4-pdg application for the Yarosh study but 1 hr after topical enzyme delivery in this study; and 5) the number of mice used in each treatment group (25 in the Yarosh design vs. 10 in the current study). Despite these considerable differences in the study design and reagents, the trends of suppressing UVB-induced carcinogenesis by topical application of DNA repair enzymes were consistent.

Overall, these data provide compelling evidence for the benefit of topical delivery of DNA repair proteins that activate the alternative repair pathways for the delay or reduction of UVB-induced carcinogenesis. Future applications of these findings in human clinical trials are anticipated to first be carried out in XP patients and in organ-transplant patients who have higher rates of NMSC.

## Methods

### Cloning, Expression, Purification, and Encapsulation of UVDE Constructs

#### Engineering UVDE-TAT and UVDE-NLS-TAT

Bacterial expression plasmids for UVDE-TAT and UVDE-NLS-TAT were created in a pET21b expression vector backbone to include a C-terminal hexahistidine tag. The UVDE construct was generated by subcloning the sequence for the *S. pombe uve1* gene (GenBank accession number NP 596165.1) between the *Nde*I and *Hin*dIII sites into the multiple cloning sequence, leaving a short linker encoding for LAAALE between the last codon for the protein (K) and the hexahistidine tag (HHHHHH). The sequence encoding the AAALE was removed and substituted with sequences to encode the TAT peptide (YGRKKRRQRRR) with the intervening DNA sequence being 5′-TAT GGC CGC AAA AAG CGC CGT CAG CGC CGT CGC-3′, to generate the UVDE-TAT expression construct. Similarly, site directed mutagenesis was used to insert the NLS-TAT sequence (PKKRKRRLYGRKKRRQRRR), encoded by 5′-CCA AAG AAG AGG AAA AGG AGG CTA TAT GGC CGC AAA AAG CGC CGT CAG CGC CGT CGC-3′, yielding the UVDE-NLS-TAT expression construct.

#### Fermentation and purification of UVDE-TAT and UVDE-NLS-TAT

Single colony isolates of BL21(DE3) with pET-UVDE-TAT or UVDE-NLS-TAT were grown overnight in 125 mL of Terrific Broth (TB) (Corning 46–055-CM, 12.0 g casein peptone, 4.0 ml glycerol, 2.31 g KH_2_PO_4_, 12.54 g K_2_HPO_4_, 24.0 g yeastolate) with 100 µg/L carbenicillin. A total of 10 mL of the overnight culture was used to inoculate a 1 L TB with 100 µg/L carbenicillin. The culture was shaken at 250 rpm at 37 °C until an OD_600_ of 4 was achieved. Protein expression was induced overnight with 0.2 mM IPTG at 16 °C. Cell paste was harvested by centrifugation and frozen at −80 °C before purification.

Cell paste was resuspended in lysis buffer (20 mM HEPES, pH 7.5, 20 mM imidazole, pH 7.5, 500 mM NaCl, 1 mM MnCl_2_, 10% glycerol, 0.5 mM PMSF, 14 mM β-mercaptoethanol) and lysed using a microfluidizer (Microfluidics, Inc., Model 110 L). The resulting lysate was cleared by centrifugation at 38,400 g for 60 min at 4 °C. The cleared lysate was applied to a 5 mL HisTrap HP column (GE Healthcare). The column was washed extensively with lysis buffer before the protein was eluted with a gradient of 20–300 mM imidazole and followed with 400 mM imidazole. Fractions containing UVDE-TAT or UVDE-NLS-TAT were pooled and loaded to a 5 mL HiTrap Heparin HP column (GE Healthcare). After extensive washes with Heparin Buffer A (25 mM HEPES pH 7.5, 500 mM NaCl, 10% glycerol, 1 mM MnCl_2_, 0.5 mM PMSF, 14 mM β-mercaptoethanol), the protein was eluted with a gradient of 500–1,000 mM NaCl. The heparin pool was concentrated and dialyzed into storage buffer (25 mM HEPES, pH 7.5, 500 mM NaCl, 1 mM MnCl_2_, 10% glycerol, 1 mM TCEP) or further purified by HiPrep 26/60 Sephacryl S-200 HR size exclusion column (GE Healthcare) in the storage buffer. The protein was concentrated using Amicon Ultra-15 centrifugal filter units (EMD Millipore), flash frozen in liquid nitrogen, and stored at −80 °C. The final yields of UVDE-TAT and UVDE-NLS-TAT were approximately 106 and 14 mg/L of *E. coli* culture, respectively. Photographs of the Coomassie-stained gels were not manipulated or spliced, but represent a single stained gel.

#### Engineering cv-pdg-NLS

In order to create a cv-pdg-NLS expression vector (pcv-pdgNLS), the coding sequence for the gene encoding cv-pdg (from the *Paramecium bursaria* Chlorella virus 1 genome, *PBCV-1*: GenBank accession number JF411744.1) was engineered to contain a six-nucleotide spacer and a sequence encoding a nuclear localization signal (NLS) between the final codon and the stop translation codon (TGA). The NLS sequence (5′-CCC GGG CCA AAG AAA AAG AGG AAG AGG CTA-3′) encodes for the amino acid sequence PGPKKKRGRL. This expression construct was cloned into pET24a between the *Nde*1 and *Hin*dIII restriction sites. The sequences of all gene constructs were verified prior to expression studies. The plasmid was transformed into BL21 (DE3) cells and glycerol stocks were made for expression and purification.

#### Large-scale fermentation and purification of cv-pdg-NLS

Fed-batch culture was carried out in a stirred tank fermenter, with a base medium of minimal R/2 medium (2 g of (NH_4_)_2_HPO_4_, 6.75 g of KH_2_PO_4_, 0.85 g of citric acid, and 0.7 g of MgSO_4_∙7H_2_O per liter) supplemented with 20 g/L yeast extract and 0.5 ml/L trace metal solution (27 g of FeCl_3_∙6H_2_O, 2 g of ZnCl_2_∙4H_2_O, 1 g of CuCl_2_, 2 g of CoCl_2_∙6H_2_O, 0.5 g of H_3_BO_3_, 1 g of CaCl_2_∙2H_2_O, and 2 g of Na_2_MoO_4_∙6H_2_O per liter of 1.2 N HCl), 8 g/L glucose, 10 g/L glycerol, 25 mg/L kanamycin, and 0.1 ml/L antifoam. An overnight seed culture was prepared from a glycerol stock of BL21 (DE3) with pET24a-cv-pdg-NLS in the supplemented R/2 medium without glycerol and antifoam. The batch phase was run at 37 °C. The pH was controlled at 7.0 by additions of 4 M NH_4_OH or 4 M H_3_PO_4_. The dissolved-oxygen concentration was controlled at 30% of air saturation (dO_2_ controller was set to cascade between agitation speed and O_2_ supplementation). When the OD_600_ reached approximately 10, a glucose feed (50% glucose, 7 g/L MgSO_4_) was initiated to maintain glucose level above 2 g/L. When the OD_600_ reached 42, the feed was switched to glycerol (50% glycerol, 7 g/L MgSO_4_) and the temperature was lowered to 19 °C over the course of about 30 min. Protein expression was induced by adding galactose to 2 g/L in one bolus. Biomass was harvested 5 hr post induction.

Cell paste was resuspended in lysis buffer (25 mM Tris-HCl, pH 8.0, 150 mM NaCl) and processed using a microfluidizer (Model M110L, Microfluidics, Inc.). The resulting lysate was cleared by centrifugation at 38,400 g for 60 min at 4 °C. The cleared lysate was applied to a Q Sepharose FF column (GE Healthcare). The Q flow-through was collected and loaded to a SP Sepharose HP column (GE Healthcare). The column was washed extensively with IEX Buffer A (25 mM Tris-HCl, pH 8.0, 150 mM NaCl) before step-elution with 0.2 M, 0.3 M, 0.4 M and 0.5 M NaCl in IEX Buffer B (25 mM Tris-HCl, pH 8.0, 0.5 M NaCl). Fractions containing cv-pdg-NLS from 0.4 M NaCl elution were pooled and further purified by size exclusion using a HiPrep 26/60 Sephacryl S-100 HR column (GE Healthcare) in 1x PBS, pH 7.4. The protein was concentrated by ultrafiltration, flash frozen, and stored at −80 °C.

#### Encapsulation of Repair Enzymes

1,2-dioleoyl-*sn*-glycero-3-phosphocholine (DOPC), 1,2-dioleoyl-*sn*-glycero-3-phosphoethanolamine (DOPE), and cholesteryl hemisuccinate (CHEMS) were purchased from Avanti Polar lipids (Alabaster, AL). Oleic acid was purchased from Sigma Aldrich (St Louis, MO). Carbomer 940 was purchased from Makingcosmetics Inc (Snoqualmie, WA). Track-etch polycarbonate membranes, engineered with pores ~2 μm, 1 μm, 400 nm and 200 nm were purchased from Millipore (Billerica, MA), Lipex liposome extruder was from Northern Lipids (Burnaby, Canada). Dermal syringes and other formulation packaging materials were purchased from Medi-Dose Inc (Warminster, PA). All common lab chemicals and reagents were from Sigma-Aldrich and Thermo Fisher Scientific (Waltham, MA).

Liposomes were composed of 2:2:5:1 molar ratio of DOPE:DOPC:CHEMS:Oleic Acid encapsulating cv-pdg-NLS, UVDE-TAT, and UVDE-NLS-TAT at 10 µg/mL each. Lipids were dissolved in chloroform and the solvent was evaporated using a Büchi (RE-121) rotary evaporator (Flawil, Switzerland) under vacuum for 4 hr. A thin lipid film was formed. The lipid film was hydrated with the repair enzymes in PBS buffer for 2 hr at 37 °C. The milky solution of liposomes was extruded consecutively 20 times through 2 μm, 1 μm, 400 nm and 200 nm polycarbonate membrane filter using a Lipex extruder connected to high pressure argon cylinder. The sizes of the liposomes were measured using Malvern Zetasizer nano ZS90 (Malvern, United Kingdom).

A total of 10 g of Carbomer 940 was added to 1000 mL of PBS to form a 1% hydrogel solution. The solution was mixed using a Gowe® Electrical Compact Laboratory mixer. The pH of the mixture was adjusted to 7.4 by slow addition of NaOH. At pH above 6 the mixture forms a gel structure. The hydrogel was mixed thoroughly for 3 hr at room temperature. Liposomes were added to 1% hydrogel solution to make the final hydrogel concentration of 0.75%. The solutions were mixed thoroughly for 1 hr at room temperature and packaged in dermal syringes.

### Animal Carcinogenesis and Tissue Preparation

Female SKH1 hairless mice (6 weeks old) were obtained from Jackson Laboratories and group-housed at 5 mice per box. All animal protocols were pre-approved through the OHSU Institutional Animal Care & Use Committee and monitored by the Department of Comparative Medicine. All methods and procedures were performed in strict accordance with the policies and specified experimental design that was previously approved by the OHSU Institutional Animal Care & Use Committee.

A total of 50 SKH1 hairless mice were randomly divided into 4 treatment groups (10 mice each), while an additional 10 mice were held with no treatments for observation of spontaneous tumor formation. After mice were housed for 2 weeks, all mice in each treatment group: 1) control empty liposome, 2) liposomes containing UVDE-TAT, 3) liposomes containing UVDE-NLS-TAT or 4) liposomes containing cv-pdg-NLS were treated as follows. One hr prior to UVB irradiation, 0.2 mL of the liposomal formulations were uniformly applied to the dorsal surface of each mouse using a pre-moistened cotton swab. This volume of liposome was absorbed within 3–5 min following application. Mice were irradiated 1 hr after application of the liposome in the morning on Monday, Wednesday, and Friday with UVB in a ventilated 8-chambered plexiglass box that was covered with a UVB-transmissible quartz plate. During the first 3 weeks with 9 total exposures, all irradiated mice were exposed to 225 J/m^2^ per exposure to allow photoaging without sunburn or blistering. Following this 3-week period of UVB acclimation, mice received ∼22 kJ/m^2^/week and all mice were examined at least three times per week for an additional 30 weeks. Although mice were monitored at least three times per week for tumor formation, the recording of tumor sizes was performed at 15, 18, 19, 20, 21, 22, 23, 24, 26, 28, 30, 32, and 33 weeks. At 23 weeks of UVB exposures, all mice were photographed for a visual record of the condition of the dorsal skin. Mice that developed either tumors >8 mm in diameter or ulcerated tumors were euthanized. Immediately following euthanasia, epidermal and dermal tissues were collected from representative tumor and non-tumor sections of the backs of the mice. Additionally, at the termination of the study (after 33 weeks), mice from the untreated group (no liposomes and no UVB treatment) were also euthanized and representative skin samples harvested. Tissues were fixed in aqueous-buffered zinc formalin (4% (w/v) formaldehyde and 600 ppm zinc) and after 2 days transferred into 70% (v/v) ethanol. Tissues were paraffin embedded and sections cut for hematoxylin and eosin (H&E) staining. Photographs were taken on a Zeiss ApoTome 2, using the Zeiss AxioCam 506 CCD color camera with no internal magnification. Scale bars were added using Image J software.

### Biostatistical Methods

The two time-to-event outcomes (death and first 2 mm tumor) were depicted graphically with nonparametric Kaplan-Meier curves for each treatment group and compared using the log-rank test. Reported log-rank p-values for pairwise comparisons between treatment groups were adjusted for multiplicity using the Tukey method^41^. Cox regression was applied to the time-to-death outcome to estimate hazard ratios for the risk of tumor-induced euthanasia when comparing each active treatment group to the control group. The Cox model assumption that the risks of death are proportional over the study period for the groups being compared was checked and found to be justifiable.

There were no missing values for total tumor size comparisons at either of the two time points of interest (23 weeks and 33 weeks) since a mouse’s last measured value was carried over if it had been euthanized prior to the time point. Although the sample distributions of total tumor size were often positively skewed, this outcome was nonetheless analyzed using a one-way ANOVA as this method is known to be robust to deviations from the normality assumption. Moreover, discrepancies in the variance of total tumor size between treatment groups discouraged the use of a nonparametric method that assumes the distributions have equal spread. At each of the two time points of interest, the significance of total tumor burden differences between treatment groups was quantified with Tukey-adjusted pairwise p-values from the corresponding ANOVA model.

Statistical significance was ascribed to all effects with multiplicity-adjusted p-values < 0.05. Statistical analysis was done in SAS 9.4 and R 3.4.
